# Community Genetics Reveal Elevated Levels of Sympatric Gene Flow among Morphologically Similar but Not among Morphologically Dissimilar Species of Lake Victoria Cichlid Fish

**DOI:** 10.4061/2011/616320

**Published:** 2011-11-24

**Authors:** N. Konijnendijk, D. A. Joyce, H. D. J. Mrosso, M. Egas, O. Seehausen

**Affiliations:** ^1^Institute of Ecology and Evolution, University of Bern, Baltzerstrasse 6, CH-3012 Bern, Switzerland; ^2^EAWAG, Center for Ecology, Evolution and Biogeochemistry (CEEB), Seestrasse 79, CH-6047 Kastanienbaum, Switzerland; ^3^Institute for Biodiversity and Ecosystem Dynamics, University of Amsterdam, Science Park 904, 1098 XH Amsterdam, The Netherlands; ^4^Department of Biological Sciences, University of Hull, Hull HU6 7RX, UK; ^5^Tanzanian Fisheries Research Institute, P.O. Box 475, Mwanza, Tanzania

## Abstract

We examined genetic structure among five species of Lake Victoria haplochromine cichlids in four island communities, using a full factorial sampling design that compared genetic differentiation between pairs of species and populations of varying morphological similarity and geographical proximity. We found that allopatric conspecific populations were on average significantly more strongly differentiated than sympatric heterospecific populations of morphologically similar species. Allopatric heterospecific populations of morphologically dissimilar species were most differentiated. Our work demonstrates that phenotypic divergence can be maintained and perhaps even evolve in sympatry despite considerable gene flow between species. Conversely, phenotypic resemblance among conspecific populations can be maintained despite geographical isolation. Additionally we show that anthropogenically increased hybridization does not affect all sympatric species evenly but predominantly affects morphologically similar and closely related species. This has important implications for the evolution of reproductive isolation between species These findings are also consistent with the hypothesis of speciation reversal due to weakening of divergent selection and reproductive isolation as a consequence of habitat homogenization and offers an evolutionary mechanistic explanation for the observation that species poor assemblages in turbid areas of the lake are characterized by just one or two species in each of a few morphologically distinct genera.

## 1. Introduction

How common gene flow is in nature among young, yet morphologically and ecologically distinct species, and how much phenotypic differentiation can be maintained in its face, is subject of considerable debate [1–3]. Investigating young adaptive radiations can be illuminating in this regard [4, 5]. In the history of life, adaptive radiations were an important source of species diversity, being bursts of speciation associated with ecological diversification often without major geographical barriers [6]. Hence, understanding the constraints to speciation and species coexistence in adaptive radiations may help understand the evolutionary structure of biodiversity more generally. The Lake Victoria cichlid species flock is one of the largest and fastest of all known adaptive radiations. With at least 500 species of cichlid fish, most of which most probably arose after the last desiccation of the lake, 15.000 years ago [7–9], this is an extraordinarily young radiation [10]. The more than 100 species of rock bottom-dwelling cichlids in Lake Victoria are defined by a combination of differences in coloration and morphological differences that often coincide with ecological differentiation. Divergence among these species is so recent that intrinsic isolation seems almost completely absent [11, 12]. 

In the similar but older Lake Malawi mbuna radiation, Rico et al. [13] found little evidence of recent gene flow among sympatric species. Genetic distances among conspecific allopatric populations were generally smaller than among sympatric heterospecific populations, and the latter were no lower than those between allopatric heterospecific populations. This suggested speciation was not very recent and was followed by range expansion with little or no gene flow in secondary sympatry. The mbuna radiation is about 0.48 million years old [14, 15], and the lake has undergone many lake level fluctuations that would separate and reunite habitat patches, perhaps permitting time for allopatric origins of rather strong reproductive barriers among some species despite considerable evidence for historical [15] and recent [16, 17] gene flow among other species. Given the very short history of the Lake Victoria radiation, allopatric origins of strong reproductive barriers would seem less likely in Lake Victoria. Samonte et al. [18] used 11 genetic markers to investigate the genetic structure between four species of the Lake Victoria species flock. These species were considerably differentiated in ecology and morphology, but Samonte et al. [18] did not find any statistically significant signal of genetic structure amongst them, leading the authors to suggest that the Lake Victoria flock may consist of one large gene pool without real species. 

**Figure 1:**
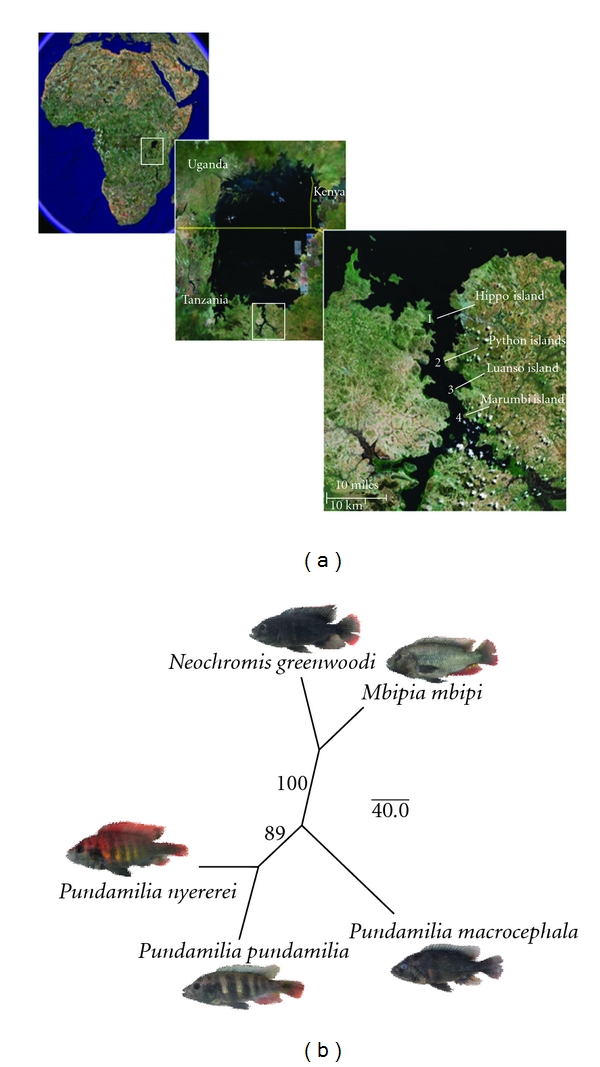
Species and sampling sites. (a) Lake Victoria, the Mwanza Gulf, and the four sampling localities, (1) Hippo Island, (2) Python Islands, (3) Luanso Island, and (4) Marumbi Island (Tanzania). (b) A neighbour joining tree for the five species investigated. Allele frequencies were estimated from AFLP data in AFLP-Surv and Reynolds genetic distance was calculated with 100 bootstrap replicates.

We studied five sympatric putative species of Lake Victoria cichlids varying in their morphological similarity and probably in their relatedness, at four locations near rocky shore islands in the Mwanza region (Tanzania, Figure 1). The Mwanza Gulf is characterized by a strong North-South gradient in water turbidity (turbid in the South, relatively clear in the North). The existence of the gradient is probably natural, or at least was already present almost 100 years ago [19], but recent anthropogenic eutrophication has intensified and steepened the gradient [20]. Putatively closely related (i.e., congeneric, where genera are morphologically defined) sympatric species are more numerous [21], and have in one case been shown to be also genetically more distinct in clearer waters in the North, but previous genetic data were restricted to microsatellite DNA in one sister species complex, *Pundamilia pundamilia *and *P. nyererei* [20]. Here, we extend this to take a community genetics approach. We used a larger number of genomic amplified fragment length polymorphisms (AFLPs) to infer phylogenomic relationships, genetic differentiation and gene flow within and between island locations among five morphologically differentiated species that coexist at all of these islands and together make the numerically dominant component of each of these communities. AFLPs have proven powerful in resolving population genetic and phylogenetic structure in cichlid fish radiations [22–26]. Fixation indices were compared between groups of population pairs representing morphologically defined clades and geographical coincidence, allowing us to compare effects of spatial proximity on morphologically similar and dissimilar taxa.

## 2. Materials and Methods

### 2.1. Sampling

Individuals of five sympatric species of rock-dwelling haplochromine cichlids were collected by angling and gill netting around four little islands in the Mwanza Gulf between February and April 2005 (Figure 1). Only males were used for this study due to the difficulty of identifying females reliably to species level for some of the species. At the locations close to Luanso and Marumbi islands where intermediate phenotypes between the blue *Pundamilia pundamilia* and the red *P. nyererei* are common, we selected typical blue and typical red males from a large sample of males. Intermediate phenotypes between *N. greenwoodi* and *M. mbipi* were also common there, and we proceeded in the same way, using the characteristics described for these two species from other islands [27] to classify individuals as *N. greenwoodi*-like and *M. mbipi-*like. Of each fish a digital picture was taken immediately after capture in a photo cuvette and fin clips were taken, preserved in 95% ethanol and stored at −20°C. We collected, preserved and identified all cichlids species caught at our sampling locations during our sampling campaign in spring 2005. This allowed us to examine community composition by quantifying species abundance.

### 2.2. Choice of Taxa

**Figure 2:**
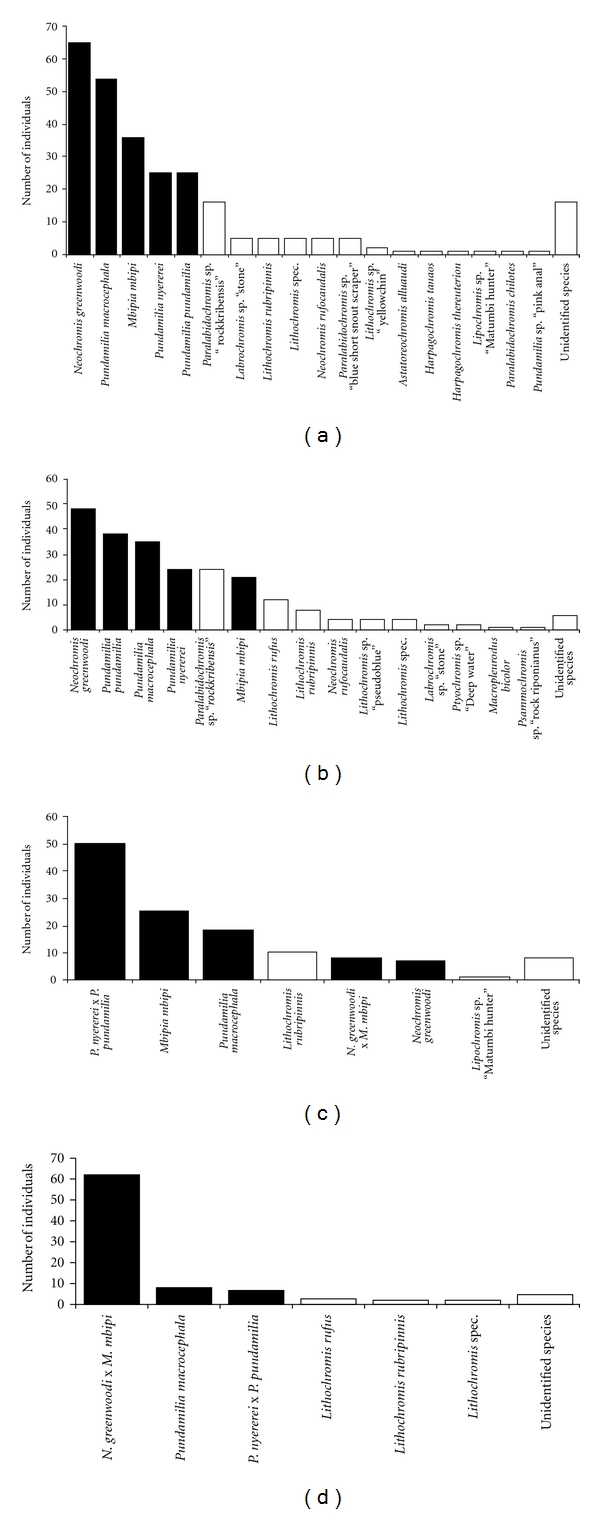
Species-abundance composition of the communities of rock-dwelling cichlids at the 4 study islands, from top to bottom: Hippo Island, Python Island, Luanso Island, and Marumbi Island. Black bars are the taxa studied in this paper. Cumulatively, they account for between 71% and 86% of the cichlids in each community.

The five species were selected because they were all present at each of the four sampling locations and represented two morphologically defined clades [27]: (1) the blue and red sister species *Pundamilia pundamilia* and *P. nyererei* and (2) the morphologically similar species pair* Neochromis greenwoodi *and *Mbipi mbipi*. Finally,* Pundamilia macrocephala* is morphologically more closely related to *P. pundamilia* and *P. nyererei* than to *M. mbipi* and *N. greenwoodi* but had never been studied genetically and closely resembles the latter two in its male nuptial coloration [27, 28]. These five species include the most abundant species in each of the four assemblages (Figure 2). The number of individuals that were genotyped for each of the five species ranged from four to eleven individuals per location (Table 1). 

### 2.3. DNA Extraction and AFLP Analysis

DNA of all samples was purified with a standard phenol-chloroform procedure. Subsequently, the AFLP method by Vos et al. [29] was used for further steps, with minor modifications involving the use of four fluorescence-labeled primer combinations (*Mse*I-CAT/*Eco*RI-AAG (green), *Mse*I-CTA/*Eco*RI-AAG (green), *Mse*I-CAT/*Eco*RI-ACC (blue), and *Mse*I-CTA/*Eco*RI-ACC (blue)). Selective amplification products were separated on a Beckman Coulter CEQ 8000 capillary system.

### 2.4. Band Calling and Binning

Traces were analyzed using the automatic binning procedure and checking each fragment by eye in the Fragment Analysis program of the CEQ 8000 software. We scored fragments between 60 and 260 base pairs to ensure that there were no erroneous bands between samples due to differential and unreliable amplification of larger alleles. AFLP fragments were scored as dominant markers that could either be absent (0) or present (1). Slope threshold was set to 5, relative peak height threshold set to 5% and the confidence level set to 95%. Maximum bin width was set to one. Loci that were fixed for the same allele in all populations were excluded from further analysis. From 4 primer pair combinations (cat-aag, cat-acc, cta-aag, and cta-acc), we obtained 654 polymorphic loci, 176, 188, 141, and 149 loci were obtained, respectively, from the different primer pairs. 19% of traces (randomly chosen across all plates) were repeated from restriction-ligation onwards and scored blind; mean repeatability was 88%. In order to obtain population genetic parameter estimates, the assumption was made that all populations were in Hardy-Weinberg equilibrium, an assumption supported by codominant marker studies conducted in parallel for many of the same populations [20, 30].

### 2.5. Phylogenetic Analysis

Allele frequencies were estimated from AFLP data assuming Hardy-Weinberg equilibrium, using a Bayesian method with nonuniform prior distribution in AFLP-Surv [31]. Reynolds et al. [32] genetic distance was calculated between (i) populations and (ii) species, with 100 bootstrap replicates. Neighbour joining trees were constructed with a randomised input order in PHYLIP [33] and consensus trees built, which were visualised in FigTree v1.3.1. To visualize conflicting phylogenetic signal among individuals, such as would be introduced by introgressive hybridization, the Nei and Li distance matrix of individuals was used to create a phylogenetic network based on the neighbour-net algorithm [34] as implemented in SplitsTree [35]. We included in the latter analysis 12 individuals of cichlid fish from Lake Edward (*Astatotilapia elegans *(*n* = 2), *A. aeneocolor *(*n* = 1), *A. sp. *“red chest” (*n* = 2), *A. sp. *“orange shoulder” (*n* = 1),* Enterochromis* sp. (*n* = 1)) and Lake Saka (Edward basin; *n* = 5) as a control for the interpretation of the distribution of phylogenetic conflict. Cichlids in the Lake Edward basin have been isolated from those in the Lake Victoria basin for at least several thousand years [36].

### 2.6. Population Genetic Analysis

**Figure 3:**
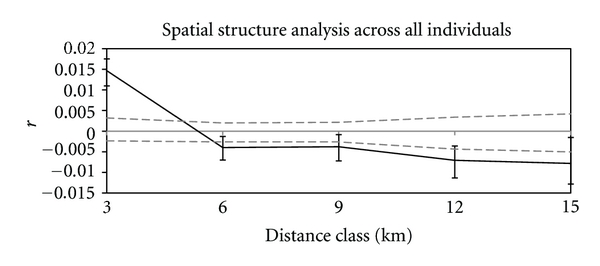
Spatial structure analysis reveals a positive autocorrelation coefficient outside the 5% and 95% confidence limits (dashed lines) among individuals within about 4 km of one another, but no isolation by distance beyond this.

Genetic differentiation of allopatric populations and of sympatric and allopatric species was estimated calculating the genetic distances as an equivalent of pairwise *F*_ST_ using the pairwise genetic distance option in Arlequin 2.0 [37] that counts the number of different alleles between haplotypes and results in weighted *F*_ST_ statistics over all loci [38, 39]. Throughout, we use *F*_ST_ as a term for this equivalent distance. We used 10 000 permutations to acquire *P* values. To test whether genetic differentiation of populations rather depended on morphological similarity or on geographical overlap, five groups of population pairs were made: (1) allopatric populations of the same morphological species, (2) sympatric populations of morphologically similar taxa that also turned out to be phylogenetic sister taxa (Figure 1), (3) allopatric populations of morphologically similar and phylogenetic sister taxa, (4) sympatric populations of morphologically dissimilar phylogenetic nonsister taxa, and (5) allopatric populations of morphologically dissimilar phylogenetic nonsister taxa. These groups correspond to those analyzed by Rico et al. [13] in Lake Malawi. Fixation indices were compared between these groups using a nonparametric Kruskal-Wallis test, because a normal distribution of *F*_ST_-equivalent values could not be assumed a priori and group sizes and variances were heterogeneous. To determine which groups differed significantly, a Mann-Whitney test was then calculated in SPSS with the Holm Sequential Bonferroni posthoc test [40] to account for multiple testing. Finally, we calculated a spatial autocorrelation coefficient for the total dataset combined, across 5 distance classes using GenAlEx [41].

## 3. Results

The species tree (Figure 1(b)) estimated from allele frequencies across all four populations of each species faithfully recovered the morphologically based classifications [27]: The morphologically similar *Neochromis greenwoodi *and *Mbipia mbipi* were phylogenetic sister taxa with high bootstrap support, and so were the morphologically similar *Pundamilia pundamilia* and *P. nyererei*, whereas *P. macrocephala* was more distantly related to the others, but somewhat closer to the other *Pundamilia* species. Since morphology and molecular markers agree remarkably well on the relationships between our five study species, we refer to them henceforth as sister and nonsister species or taxa. Note that our use of the words sister species and sister taxa is meant to reflect that these species are the most closely related among the species we sampled. We make no claim that these would remain each other's closest relatives if taxon sampling was increased within and particularly beyond our four islands. Morphological data predict that *Neochromis greenwoodi *and *Mbipia mbipi* indeed both have other closer relatives elsewhere in the lake.

Spatial autocorrelation analysis (Figure 3) demonstrated a positive autocorrelation coefficient outside the 5% and 95% confidence limits among individuals within about 4 km range of one another. When the analysis was repeated for each species individually, the same pattern held for all except *P. macrocephala*. Our islands are on average 9.2 km apart. Because of this, and since there was no gradual isolation by distance effect, our distinction between “sympatric” and “allopatric”, where the latter refer to populations living at different locations, seems appropriate. 

Our collecting effort at each of the four islands revealed that the five study species were the five numerically dominant species at each of the islands, except at Python Islands where *Paralabidochromis* sp. “rockkribensis” was more abundant than *Mbipia mbipi *(Figure 2). Together the five species accounted for between 71% and 87% of all cichlids in these assemblages, and they were the only species that occurred at each of the islands, rendering them suitable for a full factorial sampling design.

The equivalent of population-pairwise *F*_ST_ estimates for AFLP's ranged from <0.01 to 0.25 (Table 1). Negative *F*_ST_ values (3 out of 190 estimates) were set to zero. Twenty-six of the 190 pairwise fixation indices were below 0.03, with three exceptions to these were not significantly different from zero. The category of pairs with values lower than 0.03 consisted mainly of populations of the same species or of sister species, with 8 pairs of allopatric conspecific populations (8 out of 30 pairs of allopatric populations, 27%), 5 pairs of allopatric sister species (5 out of 24 pairs, 21%) and 4 pairs of sympatric sister species (4 out of 8 pairs, 50%). Fixation indices for nonsister species were less likely to be this low; that is, only 2 of 32 (6%) pairs of sympatric populations of nonsister species and 7 of 96 (7%) pairs of allopatric populations of nonsister species fell into the *F*_ST_ < 0.03 category. Of the *F*_ST_ estimates that exceeded 0.03, fourteen were not significant either. These were mostly associated with pairs in which at least one population had a sample size below six. For further analyses, we excluded populations of which we had less than six complete multilocus genotypes (3 populations were thereby excluded). 

We then had 136 estimates of pairwise fixation indices left. Among these were 21 allopatric pairs of conspecific populations, and 17 of them were significantly differentiated, implying that many of these island populations are geographically at least partially isolated. On the other hand, with one exception, sympatric populations of sister taxa were not significantly differentiated, whereas all 22 sympatric populations of nonsister taxa were. 

**Figure 4:**
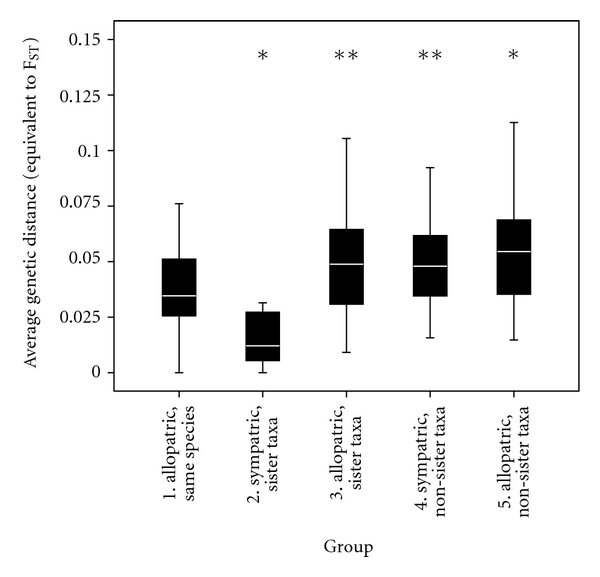
*F*_ST_ equivalent estimates for pairwise population comparisons falling into one of five different groupings: populations of the same species (always allopatric), morphologically similar taxa (*M. mbipi* and *N. greenwoodi* and *P. pundamilia* and *P. nyererei*) in sympatry and in allopatry, and nonsimilar taxa in sympatry and in allopatry. A Holm Sequential Bonferoni posteriori test was used to obtain *P* values. *Represents a significant difference against group 1, **represents a significant difference against group 2.

The Kruskal-Wallis test revealed that there were differences between the five population groupings (*P* < 0.001). Mann-Whitney tests revealed that sympatric populations of sister taxa were significantly less differentiated than any other type of populations including allopatric populations of conspecifics (Figure 4, Table 2). Sympatric populations of nonsister taxa on the other hand, were significantly more differentiated than sympatric populations of sister taxa, and very similar in their *F*_ST_ to allopatric populations of both sister and nonsister species. Allopatric populations of conspecifics were slightly, but not significantly less differentiated than allopatric populations of sister species, but they were significantly less differentiated than allopatric populations of nonsister species. Finally, sister species, when sampled in sympatry, were significantly less differentiated than when sampled in allopatry. 

**Figure 5:**
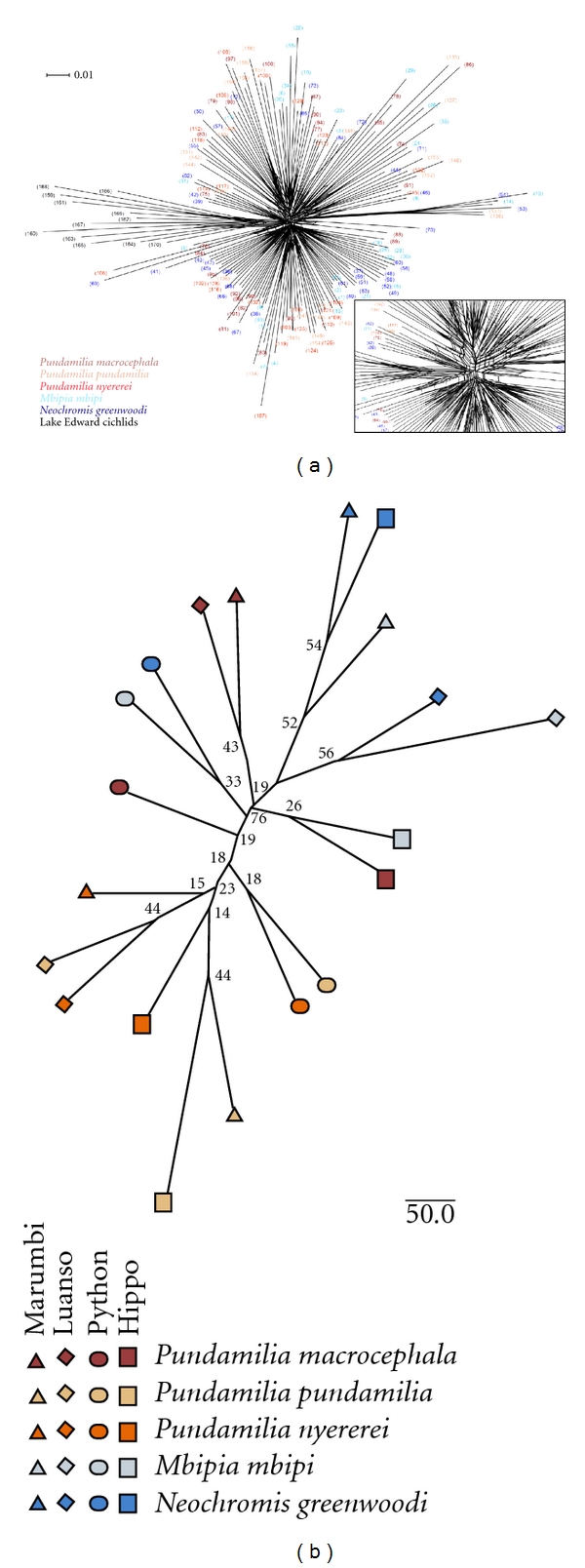
Individual-based and population trees. (a) An AFLP neighbor network based on Nei and Li distances. Samples are sorted and colour-coded by species. Conflicting phylogenetic signal in the center magnified bottom right. (b) A Neighbour joining tree for the 20 populations investigated. Allele frequencies were estimated from AFLP data in AFLP-Surv, and Reynolds genetic distance was calculated with 100 bootstrap replicates. The 4 different shapes indicate the 4 islands. The five different colours indicate the 5 species.

The individual-based neighbour net (Figure 5(a)) revealed a large amount of phylogenetic conflict about the placement of individuals of the five species and 20 populations of Lake Victoria cichlids, whereas there was somewhat less conflict about the placement of 12 individuals of Lake Edward basin cichlids relative to the taxa from Lake Victoria. The population tree (Figure 5(b)) was much better sorted than the individual-based tree but reflected the conflicting signal of phylogenetic relationship (taken here as the species tree of Figure 1) on the one hand, and local gene flow between sympatric species on the other hand. The two pairs of sister species were reciprocally monophyletic and *P. macrocephala *tended to be more distantly related to either, with signals of introgressive hybridization with *Neochromis/Mbipia *at Hippo and Python Islands. Interestingly, the abundance of *P. macrocephala *relative to *Neochromis *and *Mbipia *is much greater at these two islands than at the other two (Figure 2). Sympatric populations of sister species were in four cases each other's closest relatives, *P. pundamilia *and *P. nyererei *at Python and Luanso Islands, and *N. greenwoodi *and *M. mbipi *at the same two island locations.

## 4. Discussion

We studied five different Lake Victoria cichlid species that live on spatially isolated patches of habitat, around rocky islands. Based on morphological data [27] this set of species contains two pairs of putative sister taxa, *Pundamilia pundamilia *and *P. nyererei*, *Neochromis greenwoodi *and *Mbipia mbipi*, and one species, *Pundamilia macrocephala *that is slightly more distantly related to either. We found this morphological hypothesis to be faithfully recovered by a species tree based on 654 AFLP loci. It was also quite well recovered by a population tree based on the same loci, but there were strong signals of interspecific gene flow in sympatry, particularly between closely related species. An individual-based neighbor net indeed revealed large amounts of phylogenetic conflict, suggestive of considerable introgressive hybridization. 

Within each local species assemblage, we find good correspondence between relatedness (morphological expectations and AFLP species tree) and the AFLP-derived fixation indices. Our sample sizes per population were small, and even though this is partly compensated for by our use of a large number of loci, caution is warranted with regard to taking individual pairwise genetic distance values at face value. On the other hand, all our hypothesis tests are based on multiple replicates of pairwise comparisons from four different islands, making our main results robust to sampling error. Genetic distance between sister taxa were always lower than those between nonsister taxa, where the latter were always significant. This was true at all islands, hence in clearer waters in the northern Mwanza Gulf as much as in the very turbid waters in the South. Genetic distance between *Pundamilia macrocephala *and the species of either of the other two species pairs varied but was sometimes low and nonsignificant. Hence, at the scale of local island assemblages, *P. pundamilia* and *P. nyererei* and also *N. greenwoodi* and *M. mbipi *are indeed consistently two different pairs of population genomic sister species, and *P. macrocephala* has somewhat varying affinities to either of these taxon pairs. To the best of our knowledge, this is the first molecular genetic support for any above-species level taxonomical groupings among Lake Victoria cichlid fish. The remarkably well resolved species tree that recovers morphology-based hypotheses about relatedness among species is a definitive first in phylogenetic analyses of Lake Victoria cichlids. We suspect that our full factorial sampling design, averaging out over four islands the locally variable effects of heterospecific gene flow, may have been key to this.

Adding geographical proximity complicates the picture. Allopatric populations of the same phenotypically defined species tend to be more strongly genetically differentiated than sympatric populations of phenotypically divergent sister species. This was observed previously for the species pair *P. pundamilia *and *P. nyererei *using microsatellite markers [20], but here, we show that the same is true for a second pair of species, *Neochromis greenwoodi *and *Mbipia mbipi,* and is also often true for *Pundamilia macrocephala *against either of these other species. On the other hand, morphologically quite different species, that is, *Pundamilia pundamilia/nyererei *versus *Neochromis/Mbipia, *are genetically well differentiated independent of whether the differentiation is measured among sympatric or among allopatric populations. 

Our results differ from those obtained for a similar set of Lake Malawi rock-dwelling cichlid fish by Rico et al. [13]. Their comparisons revealed weaker genetic differentiation among allopatric populations of putative conspecifics than among sympatric populations of closely related species. Further, they found no indication that sympatric populations of closely related species were any less differentiated than allopatric populations of closely related species. From this, they concluded that there was no significant gene flow among sympatric species now or in the recent past. Rico et al. [13] proposed their analysis as a test of the hypothesis of parallel sympatric speciation within locations, which they could clearly reject with their data for the species they sampled. By the same token, our analysis of Lake Victoria cichlids would be consistent with the hypothesis of parallel sympatric speciation on our four islands. 

However, although we think parallel sympatric speciation is a possibility, perhaps a more likely explanation of our findings is that the rate of gene flow among sister species or morphologically similar species in sympatry exceeds that of gene flow between these species when they live at different islands, but also that between conspecific populations living at different islands. This situation may not be unlike that in ground finches on the Galapagos islands [5, 42], where introgressive hybridization among sympatric species is common and affects evolutionary trajectories. In either case, however, we conclude that considerable phenotypic differentiation can be maintained in Lake Victoria cichlids against a very significant amount of heterospecific gene flow. This also suggests that fairly large parts of the genome might be exchanged among species without affecting morphology in a dramatic way. Conversely, and perhaps more surprisingly, phenotypic resemblance between conspecific island populations can be maintained despite fairly little gene flow among these conspecific populations, and in fact, less than the gene flow these populations receive from sympatric heterospecifics. Speciation in Lake Victoria cichlids does not seem to happen simply as an idiosyncratic byproduct of geographical isolation, and it may indeed not even require geographical isolation on separate islands. If parallel speciation within locations was indeed explaining our observations, the phenotypic resemblance among similar species evolved in parallel on different islands would be stunning.

There are several possible alternative explanations for the differences between our study and that of Rico et al. [13]. The differences might be due to the different age of these radiations and the associated speciation events. The radiation of Lake Victoria cichlids into the current species (which is not the same as the age of the allelic variants segregating in the radiation) [36, 43] is probably one order of magnitude younger than that of the Lake Malawi Mmbuna [14, 44]. Perhaps speciation is no longer very frequent among the Lake Malawi Mmbuna, in which case reproductive isolation between species may have become stronger through time. Alternatively, the differences might be due to the particular set of Mmbuna species or the set of microsatellite loci that were investigated by Rico et al. [13]. 

Our data are also in contrast with those of Samonte et al. [18] who did not find any genetic structure among four other morphologically defined species of Lake Victoria cichlids, using 11 genetic markers. This difference may either be explained by the choice of species, or (perhaps more likely), by the number of genetic markers. Given the very short time since speciation began in Lake Victoria, and the evidence for interspecific hybridization, lineage sorting is expected to be highly incomplete and genetic differentiation among sympatric species may only reveal itself when a larger number of loci is at hand, for example, [25].

The intensity of gene flow between the species that we studied has almost certainly been affected by recent anthropogenic eutrophication, particularly in the South of our sampling region (i.e., Luanso and Marumbi islands), but even at Hippo Island water clarity has decreased. This does not invalidate any of the above discussion points. First, even though sympatric sister taxa tend to be genetically less differentiated at the southern than at the northern locations, we find even at the northern locations that sympatric sister taxa are less strongly differentiated than allopatric conspecifics and allopatric nonsister species. Second, remarkable gradients in water clarity have existed in Lake Victoria prior to anthropogenic eutrophication even though the turbid zone has recently expanded considerably into the open lake [19]. Third, paleoecological evidence suggests that since its last desiccation, Lake Victoria has undergone cycles of severe anthropogenic eutrophication and recovery on the order of thousands of years [45]. For all these reasons, we think that some gene flow between species in sympatry is most likely not just a very recent phenomenon but that the rate of hybridization has recently increased in the more turbid parts of the lake due to man-made eutrophication. However, here for the first time, we show that this anthropogenically increased rate of hybridization does not affect all sympatric species evenly, but it really affects predominantly closely related species. This has important implications for the evolution of reproductive isolation between species and its dimensionality. It is also predicted by the hypothesis of speciation reversal due to weakening of divergent selection and reproductive isolation as a consequence of habitat homogenization [46] and offers an evolutionary mechanistic explanation for the observation that the species poor assemblages in turbid areas of the lake are characterized by just one or two species in each of a few morphologically distinct genera [27].

## Figures and Tables

**Table 1:** *F*_ST_ estimates for all pairwise population comparisons (below diagonal) and corresponding *P* values (above diagonal). Significant *F*_ST_ estimates are also marked with 1 star (*P* < 0.05), 2 stars (*P* < 0.01), or 3 stars (*P* < 0.001). *F*_ST_ estimates were obtained with Arlequin 2.0. Cell colours indicate type of comparison: blue: allopatric conspecific; olive: sympatric sister species; green: allopatric sister species; yellow: sympatric nonsister species; white: allopatric nonsister species.

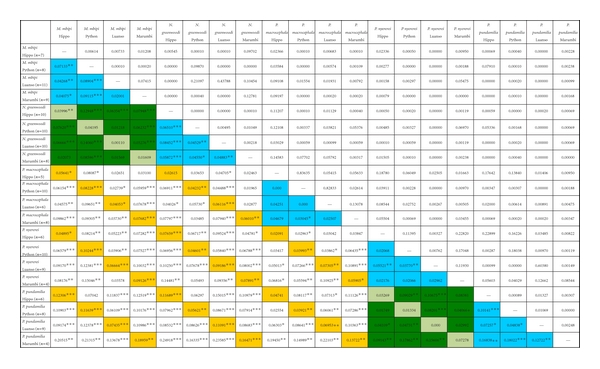

**Table 2:** Mann-Whitney test comparing *F*_ST_-equivalent estimates between populations grouped by geography and relatedness. *P* values are two tailed. Posteriori test: Holm Sequential Bonferroni (with 7 comparisons). NT: tests not performed because they are not testing predictions of our hypotheses. Results are graphically represented in Figure 4.

Groups	1	2	3	4	5
1 = Allopatric, same species	—				
2 = Sympatric, sister species	**0.008**	—			
3 = Allopatric, sister species	0.147	**0.004**	—		
4 = Sympatric, non-sister species	NT	**<0.001**	NT	—	
5 = Allopatric, non-sister species	**0.003**	NT	0.505	0.584	—
